# An Obstructing Small Bowel Phytobezoar in an Elderly Female Nigerian: A Case Report and Literature Review

**DOI:** 10.1155/2017/6962876

**Published:** 2017-06-13

**Authors:** O. S. Balogun, A. O. Osinowo, M. O. Afolayan, A. A. Adesanya

**Affiliations:** General Surgery Unit, Department of Surgery, Faculty of Clinical Sciences, College of Medicine, University of Lagos, PMB 12003, Idi Araba, Lagos, Nigeria

## Abstract

Small bowel obstruction secondary to phytobezoars is an unusual presentation in surgery. We present a case of an elderly female patient with an insidious onset of abdominal pain, abdominal distension, and bilious vomiting diagnosed radiologically to be small bowel obstruction. Exploratory laparotomy revealed a trapped mass of vegetable matter in the distal ileum. She had enterotomy with primary closure for removal of obstructing ileal phytobezoars. Her postoperative recovery was uneventful.

## 1. Introduction

Bezoar is a general term that describes entrapment of a mass of materials of different sources within the lumen of the gastrointestinal tract. Phytobezoars comprise aggregates of indigestible plant materials and mucus which can be an unusual cause of intestinal obstruction. Small intestinal obstruction due to bands, adhesions, hernias, and tumours is more commonly encountered in surgical practice.

Phytobezoars may account for about 0.4–4% of cases of acute mechanical intestinal obstruction although there is no real consensus on its exact incidence [1, 2]. Predisposing factors for phytobezoars include previous surgery on the stomach, poor mastication and edentulous jaws, rapid swallowing of large amounts fruits and vegetables, intestinal stenosis, and systemic diseases impairing gastrointestinal motility.

We present a case report of an elderly female Nigerian with normal dentition who had surgery for an obstructing small bowel phytobezoar following ingestion of large amounts of vegetable matter. We also conduct a review of literature on the management of phytobezoars.

## 2. Case Presentation and Management

78-year-old known hypertensive woman was referred from a Private Hospital to the emergency room of Lagos University Teaching Hospital with an insidious onset of colicky right lower abdominal pain of 6-day duration and recurrent copious nonprojectile bilious vomiting of 5-day duration. She last opened bowel 5 days before presentation. She had developed abdominal distension and anorexia since the onset of her symptoms. She had no history of weight loss or jaundice. There was a history of high intake of vegetables based on advice from her friends.

Examination at presentation revealed an ill-looking elderly woman with a respiratory rate of 28 cycles per minute and pulse rate of 100 beats per minute. Her blood pressure was 157/100 mmHg. Her oxygen saturation (SPO2) was 98%. Her chest was clinically clear. However, her abdomen was distended. There was right lower quadrant tenderness but no guarding or rebound tenderness. Bowel sounds were hyperactive. Rectal examination was normal. A working clinical diagnosis of intestinal obstruction secondary to possible right sided colonic tumor was made.

Her full blood count and white cell count, differentials, and platelets counts were within normal range. Her renal function was normal except for the finding of hypokalemia of 3.0 mmol/l.

**Figure 1:**
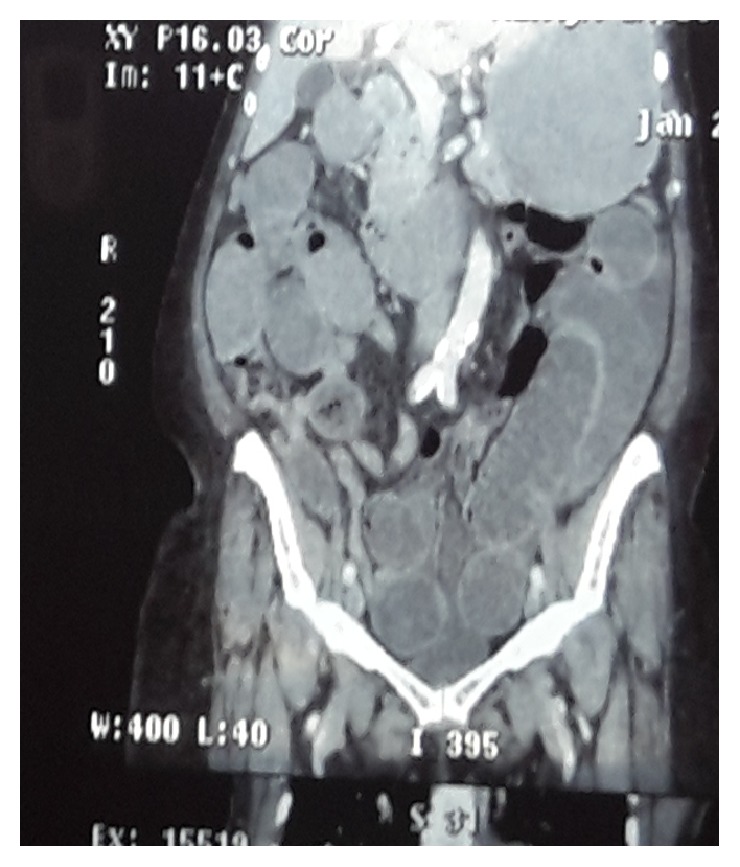
Coronal view of the computed tomography (CT) of the abdomen showing grossly dilated thickened small bowel loops up to the region of right iliac fossa.

**Figure 2:**
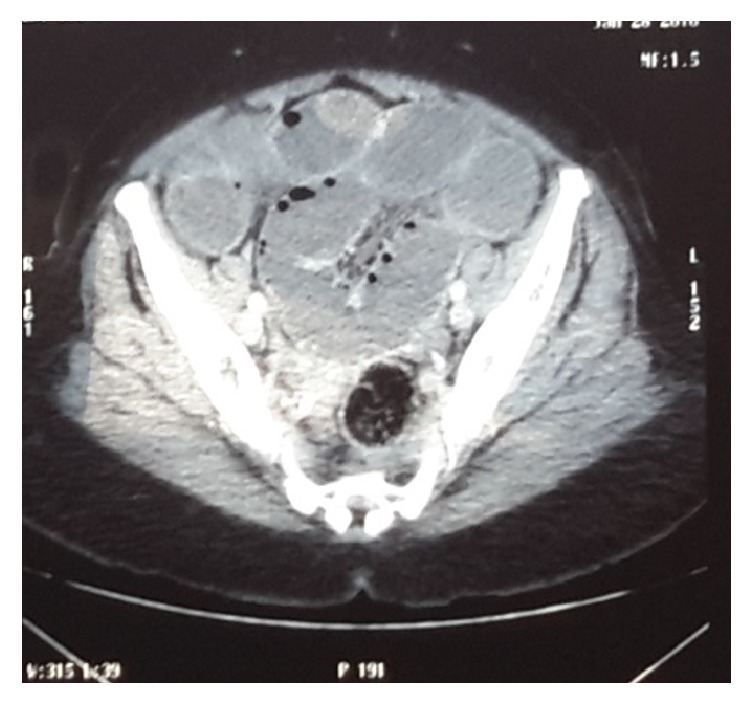
Axial view of computed tomography (CT) of the abdomen showing grossly dilated, thick-walled small bowel loops up to the region of the pelvis.

Computed tomography scan (Figures 1 and 2) revealed grossly dilated edematous small bowel loops and stomach with fluid distension. Chest X-ray showed evidence of left ventricular hypertrophy. Her electrocardiogram revealed features of sinus tachycardia, left axis deviation, and left ventricular hypertrophy.

**Figure 3:**
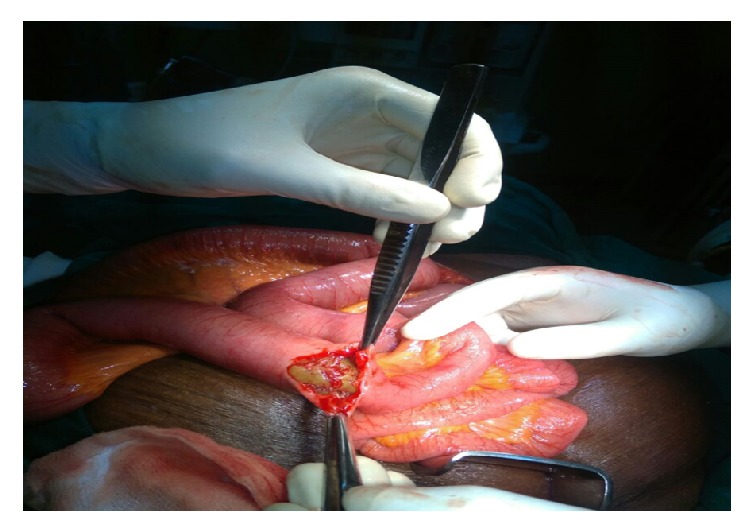
Obstructing distal ileal phytobezoar seen at laparotomy.

Patient was resuscitated and optimized for surgery. Exploratory laparotomy revealed dilated small bowel loop with a compressible obstructing mass (transition zone) at 50 cm from the ileocecal junction. The small bowel loop bowel distal to the transition zone was collapsed (Figure 3). Enterotomy of the transition zone revealed an 8 cm by 6 cm egg-shaped accretion of vegetable matter (Figure 3). Enterotomy was closed after removal of the phytobezoar. Postoperative period was uneventful. She was discharged home 5 days after surgery and was subsequently followed up at regular intervals as an outpatient.

## 3. Discussion

Mechanical intestinal obstruction in the elderly is a common presentation and indication for surgical intervention in surgery. Common intraluminal causes include tumours, gallstones, and foreign bodies. Bezoar-induced intestinal obstruction is rare. Bezoar is described according to its component and the location within the gastrointestinal tract. Four major types of bezoars include phytobezoar (derived from plant materials), trichobezoar (hair ball), lactobezoar (milk-curds), and pharmacobezoar (medications).

Gastric bezoars are found commonly in patients with poor mastication, poor gastric motility, and previous gastric surgery. Small and large bowel bezoars are uncommon. Phytobezoars are the most common bezoars encountered in surgical practice and are usually made up of concretions of plant cellulose, mucin, pectin, tannins, and mucins derived from ingested fruits and vegetables. Plant materials can aggregate to form an impacted bolus of material within the gastrointestinal tract resulting in acute intestinal obstruction. Persimmon fruit phytobezoar is the most common type reported in a series [3]. Our patient had a history of frequent ingestion of large amount of vegetable matter weeks prior to onset of her symptoms; otherwise, we found no other predisposing factors.

Clinical presentation in patients with phytobezoars depends on the type, location within the gastrointestinal tract, and presence of predisposing factors. Gastric bezoars can present with epigastric discomfort and nonspecific abdominal pains.

Small bowel phytobezoar usually presents with abdominal pain and distension, vomiting, and constipation. The principal symptoms of small bowel phytobezoars reported in a review are abdominal pain (49–100%), epigastric distress (80%), and vomiting and nausea (35–78%). Features of small bowel obstruction were found in 94.73% of cases. Less common symptoms include sensation of fullness or bloating, dysphagia, anorexia with weight loss, and even gastrointestinal hemorrhage [2, 4]. These findings support the notion that primary small bowel phytobezoar is usually obstructive in nature with average duration of 1–5 days between onset of abdominal symptoms and hospitalization [5, 6].

Diagnosing phytobezoar-induced small bowel obstruction can be challenging preoperatively. Plain abdominal X-ray showing evidence of mechanical small bowel obstruction can be seen in most patients but is not specific. Abdominal computed tomography (CT) scan is 90% sensitive and 57% specific in excluding other differential diagnoses of bezoar-induced ileus [7]. According to Zissin et al. [7], faecal matter in the small bowel on abdominal CT may appear like a bezoar. Unlike bezoar, impacted faeces tend to appear in a longer transition zone of dilated segment of small bowel proximal to the site of obstruction.

Treatment options for phytobezoar can be conservative, medical, or surgical. Conservative approach may be reasonable if the presenting symptoms resolve while the patient is being resuscitated or prepared for a more definitive treatment. Endoscopic fragmentation and extraction as well as chemical dissolution and lavage have been used in the treatment of gastric bezoars. However, surgical intervention is often required in the management of small bowel phytobezoars. Both open and laparoscopic approaches are described in the literature. Samdani et al. [8] reported a case of small bowel obstruction due to impacted apricot fruit which was successfully treated by laparoscopy. Compared to open approach, laparoscopy has benefits of less postoperative complications and shorter hospital stay. However, laparoscopy is less suitable when bowel loops are grossly dilated due to risk of enteric injury.

Definitive diagnosis of phytobezoars is often made at laparotomy. The most common site of impaction is the distal ileum at 50–70 cm proximal to ileocaecal valve [9]. This is because of relatively small diameter and reduced peristalsis of bowel content in this segment and increased water absorption [9, 10]. In our patient, obstructing phytobezoar was found at 50 cm from the ileocaecal valve.

Distal ileal phytobezoars may be fragmented manually at laparotomy and milked into the caecum [10]; more often an enterotomy with primary closure is required for definitive diagnosis and treatment [2]. Rarely, bowel resection and anastomosis are indicated for impacted bezoar with bowel ischaemia [8]. In most cases, surgical treatment of small bowel obstruction can be accomplished successfully. General preventive measures include avoidance of high fiber diet, more water consumption, proper mastication, and treatment of underlying gastrointestinal motility disorders.

## 4. Conclusion

Small bowel phytobezoar is an uncommon cause of acute intestinal obstruction in the elderly with a virgin abdomen. Preoperative aetiologic diagnosis based on history and physical examination may be difficult. Plain abdominal X-ray and ultrasound findings are that of nonspecific small bowel obstruction. Abdominal CT scan is invaluable in excluding other differential diagnoses. Surgery is often required in resolving the diagnostic puzzle and for definitive treatment. Recurrence following treatment is common and can be prevented by appropriate dietary habits and control of underlying factors.
